# Association between sleep apnea-specific hypoxic burden and severity of coronary artery disease

**DOI:** 10.1007/s11325-024-03008-1

**Published:** 2024-02-22

**Authors:** Hehe Zhang, Honghong Liu, Yuanni Jiao, Jing Zhang, Naima Covassin, Mu Wang, Yun Lin, Jiang Xie

**Affiliations:** 1 Department of Respiratory and Critical Medicine, Beijing Anzhen Hospital, Capital Medical University, #2 An Zhen Road, Beijing, 100029 China; 2 Beijing Anzhen Hospital Centre for Sleep Medicine and Science, Capital Medical University, Beijing, 100029 China; 3 Department of Cardiovascular Medicine, Beijing Anzhen Hospital, Capital Medical University, #2 An Zhen Road, Beijing, 100029 China; 4 Institute of Heart, Lung and Blood Vessel Diseases, Beijing Anzhen Hospital, Capital Medical University, Beijing, 100029 China; 5 Department of Cardiovascular Medicine, Mayo Clinic, Rochester, MN 55902 USA; 6 School of Aerospace Engineering, Tsinghua University, Beijing, 100084 China

**Keywords:** Coronary artery disease, Gensini Score, Obstructive sleep apnea, Sleep apnea-specific hypoxic burden, SYNTAX Score

## Abstract

**Purpose:**

Sleep apnea-specific hypoxic burden (SASHB) is a polysomnographic metric that comprehensively measures the degree of nocturnal desaturation caused by obstructive sleep apnea. This research was conducted to elucidate the relationship between SASHB and coronary artery disease (CAD) severity.

**Methods:**

We carried out a prospective study of hospitalized patients with CAD of unstable angina who were expected to undergo invasive coronary angiography at Beijing Anzhen Hospital from February to September 2023. SASHB values were calculated using a self-programmed C +  + program. Multivariable logistic regression analysis was applied to identify the association between SASHB and the prevalence of severe CAD, documented by the Gensini Score, and the SYNTAX (Synergy between Percutaneous Coronary Intervention With Taxus and Cardiac Surgery) Score.

**Results:**

This study enrolled 137 patients with a median age of 59 years, 96 (70.1%) of whom were male. A total of 125 (91.2%) patients had coronary stenosis of ≥ 50% in at least one location. Patients with a high SASHB of ≥ 18% min/h had a significantly higher Gensini Score (32.0 vs. 18.5, *P* = 0.002) and SYNTAX Score (14.0 vs. 7.0, *P* = 0.002) than those with a low SASHB. After adjusting for multiple covariates, a high SASHB was significantly associated with the prevalence of severe CAD, determined by a Gensini Score ≥ 21 (OR 2.67, *P* = 0.008) or a SYNTAX Score > 22 (OR 4.03, *P* = 0.016).

**Conclusion:**

Our findings revealed a robust and independent association between SASHB and CAD severity in patients with unstable angina, highlighting the potential value of SASHB as a predictor of risk and a target for interventions aimed at preventing cardiovascular diseases.

**Trial registration:**

Chinese Clinical Trial Registry No. ChiCTR2300067991 on February 2, 2023.

## Introduction

Obstructive sleep apnea (OSA), characterized by recurrent interruptions in respiration with intermittent hypoxemia during sleep, is broadly acknowledged as a contributing factor to coronary artery disease (CAD). The apnea–hypopnea index (AHI) is generated using polysomnography to describe the frequency of partial (hypopnea) and complete (apnea) breathing interruptions, allowing the determination of different degrees of OSA severity according to the number of these events. However, no significant association has been found between AHI and the severity of coronary artery lesions [1–3]. Moreover, efforts to treat OSA based on the evaluation and reduction of AHI failed to reduce the incidence of major cardiac events in large clinical trials [4, 5]. Therefore, AHI, as a measure of OSA severity, has inherent limitations and does not fully capture the cardiovascular risk associated with OSA [6]. Consequently, developing more sensitive clinical biomarkers that better reflect the close association between OSA and CAD is essential.

Hypoxia is considered the major pathogenic factor underlying OSA [7, 8]. Therefore, hypoxic indices are more powerful predictors of cardiovascular outcomes than AHI [9–12]. The sleep apnea-specific hypoxic burden (SASHB) is a score that accounts for the duration, severity, and frequency of respiratory-related desaturation events [10, 11, 13]. SASHB has been shown to be more sensitive than AHI in predicting incident heart failure and cardiovascular disease-related mortality among participants in the Sleep Heart Health Study [10] and the Osteoporotic Fractures in Men Study [11]. Although SASHB appears to be one of the most promising indices for predicting cardiovascular risk, direct evidence of the association between SASHB and structural impairment in coronary disease remains lacking. This study used the innovative algorithm of Azarbarzin et al. [11] to calculate SASHB and comprehensively evaluated its potential association with CAD severity, determined by invasive angiography.

## Study design and methods

### Study population

We enrolled individuals diagnosed with unstable angina who were hospitalized at Beijing Anzhen Hospital and scheduled for coronary angiography (and possibly subsequent percutaneous coronary intervention) from February to September 2023.The inclusion criteria were as follows: (1) availability of medical staff and research resources and (2) stable vital signs enabling overnight polysomnography. We excluded patients who met any of the following criteria: (1) acute myocardial infarction verified by cardiac enzymes and electrocardiography; (2) previous revascularization, i.e., percutaneous transluminal coronary angioplasty, percutaneous coronary intervention, or coronary artery bypass grafting; (3) severe insomnia with an Insomnia Severity Index of ≥ 22; (4) end-stage chronic diseases, such as heart failure of the New York Heart Association stage IV, advanced cancer, and renal failure on dialysis; (5) cognitive disorders or acute psychiatric episodes; (6) previous treatment with positive airway pressure for OSA or other disorders within the last 6 months; and (7) use of nocturnal oxygen supplementation that could not be withheld during sleep test. This study was approved by the Institutional Review Board of Beijing Anzhen Hospital, with each patient providing written informed consent.

### Overnight polysomnography

Unattended overnight polysomnography was performed using the Alice PDx portable sleep diagnostic system, manufactured by Philips Respironics, located in Murrysville, PA, USA. Bedtime was set between 10:00 PM and 6:00 AM on the night prior to coronary angiography. Electroencephalography, electromyography, electrooculography, electrocardiography, airflow through nasal pressure and thermistor, snore microphone, pulse oximetry, and thoracic-abdominal respiratory inductance plethysmography were performed. Registered polysomnographic technologists scored the sleep stages, respiratory events, desaturation events, and arousals. Apnea was noted when a ≥ 90% reduction in airflow occurred for at least 10 s, while hypopnea referred to a ≥ 30% decrease in airflow for at least 10 s with a ≥ 3% desaturation or microarousal [14]. The total number of both apnea and hypopnea incidents was divided by the sleep duration to calculate AHI, and an AHI of ≥ 15 events/h was indicative of OSA. The oxygen desaturation index (ODI) was defined as the hourly frequency of the pulsus oxygen saturation drop of ≥ 3% from baseline. The mean oxygen saturation during sleep (meanSpO_2_), lowest oxygen saturation during sleep (minSpO_2_), and percentage of sleep time with oxygen saturation < 90% (T90SpO_2_) were directly derived from a polysomnography report. The Epworth Sleepiness Scale, Insomnia Severity Index, Zung Self-rating Anxiety Scale, Zung Self-rating Depression Scale, and Berlin Questionnaire were administered before polysomnography [15–18]. Morning blood pressure and heart rate were measured while sitting for 10 min after getting up from bed.

### SASHB calculation

The SASHB value was determined using the C +  + program (compiled in Microsoft Visual Studio Premium 2012, version 11.0.50727.1 RTMREL) based on the innovative algorithm of Azarbarzin et al. [11]. This technique involved computing the area under each respiratory event-related oxygen desaturation curve, considering both the time span and depth of each desaturation event. The beginning and end points of each curve were identified using a search window. This window was defined by aligning all the curves of respiratory event-related oxygen desaturation with the endpoint of each respiratory event marked as time 0. The area under each respiratory-related desaturation curve within the specific search window was then summed and divided by the overall sleep duration (measured in hours) to obtain the SASHB value.

### Assessment of coronary artery stenosis

Invasive coronary angiography with iodine contrast was performed to detect coronary stenosis and occlusion. Without access to polysomnographic data, two certified cardiologists independently calculated both the Gensini Score and SYNTAX (Synergy between Percutaneous Coronary Intervention With Taxus and Cardiac Surgery) Score. These scores assess both the severity of coronary lesions and the significance of affected areas in the coronary circulation. The Gensini Score was calculated manually following an available algorithm [19], while the SYNTAX Score was determined using an online calculator [20]. Higher scores indicate more severe coronary lesions in both evaluation systems.

### Statistical analysis

Continuous variables were reported using the median along with the interquartile range (25th and 75th percentiles) owing to their skewed distributions, as observed using the Shapiro-Wilk test for normality. Dichotomous variables were reported as frequencies (percentages). Wilcoxon tests were used to compare continuous data, while Pearson’s *χ*^2^ tests were applied for the comparison of dichotomous data. To further investigate the effect of disordered breathing on CAD, we used a median split to group participants based on the median values of SASHB (18% min/h), meanSpO_2_ (94%), and T90SpO_2_ (0.6% rounded to 1%), together with the conventional cut-offs of ODI (15 events/h) and minSpO_2_ (85%). Patients with a Gensini Score or a SYNTAX Score above the median value were classified as having severe CAD. Furthermore, a SYNTAX Score of > 22, which is a conventional criterion [21], was also used to define severe CAD. A multivariate logistic regression analysis was performed to investigate the associations between sleep indices and severe CAD. Model 1 adjusted for age and sex, while Model 2 further incorporated the major risk factors for CAD, such as smoking status, hypertension, hemoglobin A_1c_, and low-density lipoprotein cholesterol (LDL) [22]. To investigate the potential dose–response relationship between SASHB and CAD severity, multiple linear regression analysis was conducted, incorporating significant variables identified in univariate regression model (log SASHB and hemoglobin A1c), major demographic variables (age and sex), and widely recognized risk factors for CAD (smoking, hypertension, and LDL). SASHB underwent log-transformation due to its skewed distribution in the regression analysis. Data analyses were performed with JMP version 16, and statistical significance was defined by a two-sided *P*-value of < 0.05.

## Results

### Basic characteristics of study patients

This study included 137 patients with a median age of 59 years, of whom 96 (70.1%) were male. Among these patients, 71 (51.8%) were diagnosed with OSA by polysomnography. Furthermore, 72 (52.6%) of the patients underwent percutaneous coronary intervention, while 16 (11.7%) only had percutaneous transluminal coronary angioplasty, and 10 (7.3%) were referred for coronary artery bypass grafting after angiography. Twelve (8.8%) patients had arterial stenosis of < 50% in the coronary tree. Despite the OSA group having a higher body mass index, neck circumference, and waist circumference, the demographic and clinical data were comparable between the OSA and non-OSA groups, as detailed in Table 1.

**Table 1:** Characteristics of the study population

Basic characteristics	All (*N* = 137)	OSA (*N* = 71)	Non-OSA (*N* = 66)	*P* value
Age, year	59 (53–65)	59 (53–67)	58 (53–64)	.182
Male sex, *N* (%)	96 (70.1)	47 (66.2)	49 (74.2)	.304
Body mass index, kg/m^2^	26.4 (24.5–28.4)	26.8 (25.7–29.7)	25.8 (23.6–27.7)	.013
Smoking^*^, *N* (%)	70 (51.1)	37 (52.1)	33 (50.0)	.805
Neck circumference, cm	39.5 (37.0–42.1)	41.0 (37.4–43.0)	39.0 (36.0–40.5)	.008
Waist circumference, cm	97.0 (91.0–104.0)	101.0 (94.0–106.0)	95.0 (88.5–99.0)	.002
Hypertension, *N* (%)	93 (67.9)	49 (69.0)	44 (66.7)	.769
Stroke, *N* (%)	8 (5.8)	5 (7.0)	3 (4.6)	.534
Diabetes, *N* (%)	45 (32.8)	23 (32.4)	22 (33.3)	.907
Hemoglobin A_1c_, %	6.1 (5.7–7.0)	6.1 (5.7–7.1)	6.1 (5.8–7.0)	.791
Left ventricular ejection fraction, %	65 (62–67)	64 (61–66)	65 (63–67)	.113
Total cholesterol, mmol/L	4.2 (3.6–5.0)	4.2 (3.6–5.0)	4.2 (3.6–5.2)	.770
Low-density lipoprotein cholesterol, mmol/L	2.3 (1.7–3.2)	2.4 (1.8–3.0)	2.3 (1.7–3.3)	.834
High-density lipoprotein cholesterol, mmol/L	1.1 (1.0–1.3)	1.1 (0.9–1.3)	1.2 (1.0–1.4)	.076
Triglycerides, mmol/L	1.6 (1.2–2.2)	1.7 (1.2–2.2)	1.5 (1.1–2.1)	.293

Data are presented as median (25th and 75th percentiles) or frequency (percentage). *OSA* = obstructive sleep apnea

^*^Smoking: current or previous smoking

Table 2 presents the sleep variables of the study patients categorized according to the diagnosis of OSA. Patients with OSA had heart rate and morning blood pressure comparable to those of the non-OSA population. Most of the patients had a low prevalence of somnolence, insomnia, anxiety, and depression. The Berlin Questionnaire accurately identified 46 (64.8%) patients with OSA before polysomnography.

**Table 2:** Polysomnographic data of study patients

Polysomnographic data	All (*N* = 137)	OSA (*N* = 71)	Non-OSA (*N* = 66)	*P* value
AHI, events/h	15 (8–28)	27 (20–40)	7 (3–11)	< .001
ODI, events/h	15(8–27)	27 (19–40)	8 (3–11)	< .001
MeanSpO_2_, %	94 (93–95)	94 (92–95)	95 (94–95)	< .001
MinSpO_2_, %	86 (81–89)	83 (77–87)	89 (87–90)	< .001
T90SpO_2_, %	0.6 (0–4.0)	2.8 (0.7–11.0)	0.1 (0–0.4)	< .001
SASHB, % min/h	18 (8–38)	37 (24–73)	8 (4–13)	< .001
Systolic blood pressure, mmHg	127 (117–139)	130 (117–141)	125 (113–136)	.057
Diastolic blood pressure, mmHg	82 (75–90)	82 (75–91)	81 (74–88)	.173
Heart rate, beat per min	67 (58–72)	66 (56–73)	67 (60–72)	.539
Epworth Sleepiness Scale	7 (4–11)	7 (4–12)	8 (5–9)	.548
Insomnia Severity Index	4 (2–8)	4 (2–8)	5 (2–8)	.450
Zung Self-rating Anxiety Scale	40 (36–43)	41 (36–44)	39 (36–43)	.209
Zung Self-rating Depression Scale	43 (37–46)	42 (38–45)	43 (36–46)	.337
High risk of OSA by Berlin Questionnaire	69 (50.4)	46 (64.8)	23 (34.9)	< .001

Data are presented as median (25th and 75th percentiles) or frequency (percentage). *AHI* = apnea hypopnea index; *meanSpO2* = mean oxygen saturation during sleep; *minSpO*_*2*_ = lowest oxygen saturation during sleep; *ODI* = oxygen desaturation index; *OSA* = obstructive sleep apnea; *SASHB* = sleep apnea-specific hypoxic burden; *T90SpO*_*2*_ = percentage of sleep time with oxygen saturation < 90%

### Sleep apnea and distribution of coronary lesions

Patients with OSA had a higher occurrence of ≥ 50% stenosis in the right coronary artery than those without OSA (62.0% vs. 36.4%, *P* = 0.003). Similarly, the prevalence of ≥ 50% stenosis in the right coronary artery was greater in patients with higher values of SASHB than those with lower SASHB values (63.8% vs. 35.3%, *P* < 0.001) (Table 3). However, the investigation revealed no significant differences in the distribution of ≥ 50% stenosis in the left main-left anterior descending branch and left circumflex branch between the studied sub-groups.

**Table 3:** Prevalence of ≥ 50% coronary stenosis according to OSA characteristics

	Left main-left anterior descending branch	Right coronary artery	Left circumflex branch
OSA (*N* = 71)	52 (73.2)	44 (62.0)	35 (49.3)
Non-OSA (*N* = 66)	47 (71.2)	24 (36.4)	24 (36.4)
*P* value	.791	.003	.127
High SASHB (*N* = 69)	53 (76.8)	44 (63.8)	34 (49.3)
Low SASHB (*N* = 68)	46 (67.7)	24 (35.3)	25 (36.8)
*P* value	.231	< .001	.139
High ODI (*N* = 73)	55 (75.3)	46 (63.0)	37 (50.7)
Low ODI (*N* = 64)	44 (68.8)	22 (34.4)	22 (34.4)
*P* value	.390	< .001	.054
High minSpO_2_ (*N* = 75)	50 (66.7)	30 (40.0)	27 (36.0)
Low minSpO_2_ (*N* = 62)	49 (79.0)	38 (61.3)	32 (51.6)
*P* value	.108	.013	.066
High meanSpO_2_ (*N* = 58)	38 (65.5)	30 (51.7)	26 (44.8)
Low meanSpO_2_ (*N* = 79)	61 (77.2)	38 (48.1)	33 (41.8)
*P* value	.131	.675	.721
High T90SpO_2_ (*N* = 59)	47 (79.7)	37 (62.7)	28 (47.5)
Low T90SpO_2_ (*N* = 78)	52 (66.7)	31 (39.7)	31 (39.7)
*P* value	.093	.008	.367

Data are presented as frequency (percentage). *AHI* = apnea hypopnea index; *meanSpO*_*2*_ = mean oxygen saturation during sleep; *minSpO*_*2*_ = lowest oxygen saturation during sleep; *ODI* = oxygen desaturation index; *OSA* = obstructive sleep apnea; *SASHB* = sleep apnea-specific hypoxic burden; *T90SpO*_*2*_ = percentage of sleep time with oxygen saturation < 90%

### Sleep disorder and CAD severity

Patients with OSA had more severe coronary lesions than those without OSA, determined by the Gensini Score (28.0 vs. 20.0, *P* = 0.020) and SYNTAX score (13.0 vs. 8.0, *P* = 0.021) (Table 4). Patients with high SASHB had more severe coronary lesions than those with low SASHB (Gensini Score: 32.0 vs. 18.5, *P* = 0.002; SYNTAX Score: 14.0 vs. 7.0, *P* = 0.002). Similarly, patients with a higher ODI, lower minSpO_2_, and higher T90SpO_2_ exhibited a higher Gensini Score and SYNTAX Score than their counterparts.

**Table 4:** Severity of CAD according to OSA characteristics

Data	Gensini Score	SYNTAX Score
OSA (*N* = 71)	28.0 (9.0–57.0)	13.0 (7.0–22.5)
Non-OSA (*N* = 66)	20.0 (6.0–33.0)	8.0 (4.8–14.3)
*P* value	.020	.021
High SASHB (*N* = 69)	32.0 (11.5–56.0)	14.0 (8.0–21.3)
Low SASHB (*N* = 68)	18.5 (6.0–29.8)	7.0 (4.0–13.8)
*P* value	.002	.002
High ODI (*N* = 73)	28.0 (11.5–59.0)	13.0 (7.0–23.3)
Low ODI (*N* = 64)	19.5 (5.3–29.8)	7.5 (4.0–13.8)
*P* value	.002	.002
High minSpO_2_ (*N* = 75)	19.0 (5.0–32.0)	8.0 (4.0–13.0)
Low minSpO_2_ (*N* = 62)	32.0 (16.5–58.0)	15.0 (7.8–25.3)
*P* value	< .001	< .001
High meanSpO_2_ (*N* = 58)	22.5 (6.0–40.0)	9.0 (4.0–16.3)
Low meanSpO_2_ (*N* = 79)	20.0 (8.0–44.0)	13.0 (7.0–20.0)
*P* value	.655	.064
High T90SpO_2_ (*N* = 59)	28.0 (12.0–57.0)	15.0 (8.0–24.0)
Low T90SpO_2_ (*N* = 78)	20.0 (5.0–36.5)	8.0 (4.0–13.3)
*P* value	.004	.001

Data are presented as median (25th and 75th percentiles). *AHI* = apnea hypopnea index; *CAD* = coronary artery disease; *meanSpO*_*2*_ = mean oxygen saturation during sleep; *minSpO*_*2*_ = lowest oxygen saturation during sleep; *ODI* = oxygen desaturation index; *OSA* = obstructive sleep apnea; *SASHB* = sleep apnea-specific hypoxic burden; *SYNTAX* = Synergy between Percutaneous Coronary Intervention with Taxus and Cardiac Surgery; *T90SpO*_*2*_ = percentage of sleep time with oxygen saturation < 90%

### Association of SASHB with the prevalence of severe CAD

A significant association was shown between high SASHB levels and the prevalence of severe CAD, defined by the Gensini Score and SYNTAX Score greater than their respective median values of 21 and 11 (OR 2.51, 95% CI 1.26 − 5.00, *P* = 0.009 and OR 3.23, 95% CI 1.60 − 6.50, *P* = 0.001) (Table 5). This association persisted after adjusting for demographic factors for age and sex in Model 1 (OR 2.56, 95% CI 1.28 − 5.13, *P* = 0.008 and OR 3.17, 95% CI 1.56 − 6.47, *P* = 0.002), and for six major risk factors for CAD (age, sex, smoking, hypertension, hemoglobin A_1c_, and LDL) in Model 2 (OR 2.67, 95% CI 1.30 − 5.48, *P* = 0.008 and OR 3.83, 95% CI 1.77 − 8.30, *P* < 0.001). When modeling SASHB as a continuous variable, an increase of 1-U in SASHB led to approximately 2% higher odds of having severe CAD in univariate (OR 1.02, 95% CI 1.01 − 1.03, *P* = 0.006) and in adjusted Model 1 and Model 2 (OR 1.02, 95% CI 1.00 − 1.03, *P* = 0.007 and OR 1.02, 95% CI 1.00 − 1.03, *P* = 0.014). Furthermore, when stratifying patients according to the conventional criterion of severe CAD (SYNTAX Score > 22), patients with high SASHB also showed higher odds of having severe CAD in adjusted Model 2 (OR 4.03, 95% CI 1.30 − 12.47, *P* = 0.016).

**Table 5:** Association of SASHB with severe CAD as identified by Gensini Score and SYNTAX Score

Severe CAD	Univariate model			Adjusted model 1^a^			Adjusted model 2^b^		
	OR	95% CI	*P* value	OR	95% CI	*P* value	OR	95% CI	*P* value
Gensini Score ≥ 21									
SASHB, per 1 unit	1.01	1.00–1.02	.094	1.01	1.00–1.02	.100	1.01	1.00–1.02	.064
High SASHB vs low SASHB	2.51	1.26–5.00	.009	2.56	1.28–5.13	.008	2.67	1.30–5.48	.008
SYNTAX Score ≥ 11									
SASHB, per 1 unit	1.02	1.01–1.03	.006	1.02	1.00–1.03	.007	1.02	1.00–1.03	.014
High SASHB vs low SASHB	3.23	1.60–6.50	.001	3.17	1.56–6.47	.002	3.83	1.77–8.30	< .001
SYNTAX Score > 22									
SASHB, per 1 unit	1.00	1.00–1.01	.553	1.00	1.00–1.01	.559	1.00	1.00–1.01	.434
High SASHB vs low SASHB	2.45	0.98–6.14	.056	2.51	1.00–6.33	.051	4.03	1.30–12.47	.016

*AHI* = apnea hypopnea index; *CAD* = coronary artery disease; *CI* = confidence interval; *OR* = odds ratio; *OSA* = obstructive sleep apnea; *SASHB* = sleep apnea-specific hypoxic burden; *SYNTAX* = Synergy between Percutaneous Coronary Intervention with Taxus and Cardiac Surgery

^a^Adjusted for age and sex

^b^Adjusted for age, sex, smoking, hypertension, hemoglobin A_1c_, and low-density lipoprotein cholesterol

The multiple linear regression analysis revealed a significant association between log SASHB and the values of Gensini Score (*β* = 5.99, *P* = 0.001) and SYNTAX Score (*β* = 1.90, *P* = 0.005), with age, sex, hemoglobin A1c, smoking, hypertension, and LDL all incorporated as variables in the model (Table 6).

**Table 6:** Predictors of CAD severity by multiple linear regression analysis

Predictors^a^	Model 1 adjusted		Model 2 adjusted		Model 3 adjusted		Model 4 adjusted		Model 5 adjusted		Model 6 adjusted	
	*β*	*P* value	*β*	*P* value	*β*	*P* value	*β*	*P* value	*β*	*P* value	*β*	*P* value
Log SASHB	6.36	< .001	6.37	< .001	6.32	< .001	6.15	< .001	6.21	< .001	5.99	.001
Hemoglobin A_1c_	3.56	.049	3.59	.050	3.56	.053	3.67	.046	3.69	.046	3.47	.060
Age			− 0.03	.889	0.01	.960	0	.988	0.02	.930	0	.993
Male sex					1.12	.663	− 0.46	.877	− 0.37	.901	− 0.33	.910
Smoking							2.74	.287	2.70	.295	2.89	.261
Hypertension									− 0.70	.771	− 0.27	.910
LDL											3.61	.112
Predictors^b^	*β*	*P* value	*β*	*P* value	*β*	*P* value	*β*	*P* value	*β*	*P* value	*β*	*P* value
Log SASHB	1.97	.003	1.94	.003	1.92	.004	1.91	.004	1.93	.004	1.90	.005
Hemoglobin A_1c_	2.19	.001	2.05	.003	2.04	.003	2.05	.003	2.06	.003	2.02	.004
Age			0.13	.145	0.15	.130	0.15	.134	0.16	.126	0.16	.138
Male sex					0.44	.644	0.27	.805	0.31	.782	0.31	.778
Smoking							0.29	.759	0.28	.772	0.31	.750
Hypertension									− 0.31	.732	− 0.24	.791
LDL											0.57	.504

*CAD* = coronary artery disease; *LDL* = low-density lipoprotein cholesterol; *SASHB* = sleep apnea-specific hypoxic burden; *SYNTAX* = Synergy between Percutaneous Coronary Intervention with Taxus and Cardiac Surgery

^a^Predictors of CAD severity evaluated by Gensini Score

^b^Predictors of CAD severity evaluated by SYNTAX Score

## Discussion

This study provides evidence of a robust correlation between SASHB levels and the structural severity of CAD in individuals subjected to invasive coronary angiography. Our findings highlight the need to accelerate the application of SASHB in cardiovascular community.

Our results are consistent with those of previous studies demonstrating that SASHB is a more sensitive indicator than AHI for predicting adverse cardiovascular outcomes [10, 11]. Although AHI is a universally accepted metric for stratifying the severity of OSA, it has inherent limitations and does not reflect several key pathophysiological mechanisms activated by aberrant respiratory events during sleep. For example, AHI does not account for the duration of respiratory events or the severity of subsequent episodes of desaturation. By analyzing the frequency, duration, and depth of respiratory-related desaturation, the SASHB proved to be a robust tool for assessing OSA severity. Additional investigations should be undertaken to examine the link between SASHB and other previously identified OSA phenotypes, such as low arousal threshold, excessive sleepiness, and rapid eye movement-dominant OSA subtypes. In addition to CAD, it is important to validate the predictive value of SASHB in other cardiovascular diseases, such as hypertension, atrial fibrillation, and aortic dissection. Although SASHB has its merits, it is not without limitations. For example, SASHB does not consider an individual’s ability to endure hypoxia, as some patients may tolerate hypoxia better than others. Therefore, it is advisable to interpret the results of the SASHB in conjunction with other clinical data to obtain a more comprehensive understanding of an individual’s sleep.

To our knowledge, this study represents the first attempt to elucidate the links between SASHB levels and coronary structural impairment as measured by the Gensini Score and SYNTAX Score, both of which are prognostically relevant algorithms [23–25]. However, based on these cross-sectional results, we cannot directly conclude that SASHB is associated with cardiovascular outcomes, which has been partially confirmed in other studies on heart failure and cardiovascular fatality [10, 11]. Longitudinal studies are warranted to observe the development of coronary lesions in patients with high SASHB levels and other OSA phenotypes.

Computation of SASHB based on current polysomnography remains challenging and presently no standardized operating procedure is available. Azarbarzin et al. devised a unique search window for each subject, facilitating the computation of the area under the desaturation curve, even in cases where desaturation did not show an unambiguous beginning and end. Although the subject-specific search window strategy is convenient and practical, it has certain limitations. For example, long desaturation events may extend the search window, leading to the omission of SASHB values outside the window. In contrast, spontaneous desaturation not caused by respiratory events may still be included if they fall within the window, resulting in an overestimation of the SASHB values. Therefore, more refined techniques are required to accurately calculate the SASHB value and fulfill its promising clinical potential.

### Strengths and limitations

Direct assessment of the severity of CAD using angiography is a strength of our research. However, the study design had certain limitations. All participants were hospitalized for angina; thus, the clinical value of the SASHB could not be extrapolated to the general population. Notably, a cohort study that recruited community residents for osteoporosis research concluded that SASHB had cardiovascular prognostic significance [11]. Second, the cross-sectional study design precluded establishing a temporal association between SASHB and the development of severe CAD. A causal relationship would require confirmation through repeated coronary angiography during the follow-up assessment.

## Conclusion

Our study findings reveal that individuals with unstable angina who have high SASHB levels may show more severe coronary lesions, as observed by invasive angiography. Further studies are necessary to verify the detrimental effects of high SASHB levels on the development of CAD. Additionally, improvements in the algorithm used to calculate SASHB scores may facilitate their clinical utility.

## Data Availability

The datasets created and/or analyzed in this study are accessible from the corresponding author.
